# Implementing 360° Quantified Self for childhood obesity: feasibility study and experiences from a weight loss camp in Qatar

**DOI:** 10.1186/s12911-017-0432-6

**Published:** 2017-04-13

**Authors:** Luis Fernandez-Luque, Meghna Singh, Ferda Ofli, Yelena A Mejova, Ingmar Weber, Michael Aupetit, Sahar Karim Jreige, Ahmed Elmagarmid, Jaideep Srivastava, Mohamed Ahmedna

**Affiliations:** 1 Qatar Computing Research Institute, Hamad bin Khalifa University, HBKU Research Complex, Qatar Foundation, Education City, Doha, Qatar; 2 Department of Human Nutrition, College of Health Sciences, Qatar University, Doha, Qatar

**Keywords:** Quantified Self, eHealth, Wearable

## Abstract

**Background:**

The explosion of consumer electronics and social media are facilitating the rise of the Quantified Self (QS) movement where millions of users are tracking various aspects of their daily life using social media, mobile technology, and wearable devices. Data from mobile phones, wearables and social media can facilitate a better understanding of the health behaviors of individuals. At the same time, there is an unprecedented increase in childhood obesity rates worldwide. This is a cause for grave concern due to its potential long-term health consequences (e.g., diabetes or cardiovascular diseases). Childhood obesity is highly prevalent in Qatar and the Gulf Region. In this study we examine the feasibility of capturing quantified-self data from social media, wearables and mobiles within a weight lost camp for overweight children in Qatar.

**Methods:**

Over 50 children (9–12 years old) and parents used a wide range of technologies, including wearable sensors (actigraphy), mobile and social media (WhatsApp and Instagram) to collect data related to physical activity and food, that was then integrated with physiological data to gain insights about their health habits.

In this paper, we report about the acquired data and visualization techniques following the 360° Quantified Self (360QS) methodology (Haddadi et al., ICHI 587–92, 2015).

**Results:**

360QS allows for capturing insights on the behavioral patterns of children and serves as a mechanism to reinforce education of their mothers via social media. We also identified human factors, such as gender and cultural acceptability aspects that can affect the implementation of this technology beyond a feasibility study. Furthermore, technical challenges regarding the visualization and integration of heterogeneous and sparse data sets are described in the paper.

**Conclusions:**

We proved the feasibility of using 360QS in childhood obesity through this pilot study. However, in order to fully implement the 360QS technology careful planning and integration in the health professionals’ workflow is needed.

**Trial Registration:**

The trial where this study took place is registered at ClinicalTrials.gov on 14 November 2016 (NCT02972164).

## Background

Childhood obesity is a growing epidemic worldwide [1, 2]. Rapid societal transformations in the Gulf Co-operation Council (GCC) region, which includes Qatar, has led to a commensurate rise in lifestyle related diseases and increased rates of childhood obesity. Unhealthy habits such as eating high caloric fast food, and a sedentary lifestyle starts at an early age and can have long term consequences. New technologies can help in tackling childhood obesity by providing tools to monitor and analyze the lifestyle factors, influencing an individual’s health and well-being, and providing a new channel for healthcare interventions.

In this paper we present the experiences in deploying the 360° Quantified Self (360QS) system, a concept we introduced in [3], within a clinical study for tackling childhood obesity in Qatar [4]. 360QS is defined as a quantified-self approach providing a holistic view of the user, by integrating rich information about an individual’s health, including 1) health factors (e.g. social influences) gathered from social media, 2) physical activity sensed from mobile apps, 3) body sensing data from wearable activity tracker, 4) food image analysis and 5) general health data such as weight, height, or body composition. We must notice that the 360QS approach as defined by Haddadi et al. [3] also involves the capturing of data from individuals to help the decision making of both professionals and individuals. Although our approach is not focused on individuals who have decided to quantify themselves, we do focus on a health intervention where the individuals can take active role in capture information about their health.

360QS data can be extremely valuable since lifestyle related factors are often hard to acquire in a clinical encounter. Hence, 360QS data can provide a holistic view of the patients’ context that has health implications, in order to facilitate the decision making of patients, caregivers and healthcare professionals. In this feasibility study we also explored how human factors, in particular gender differences, have a major influence on technology acceptance and data collection.

### Objectives

Traditionally, Quantified Self technologies have been used by empowered health consumers, which might not be the more representative type of individuals in need of behavioral change support. In our study we wanted to explore the feasibility of capturing health-related information using quantified self-technologies by overweight children and their families.

Our study is based on the Technology Acceptance Model [5], but instead of focusing on ”Perceived Usefulness”,”Perceived Easy of Use”,”Attitude Towards Using” or ”Behavioral Intention to Use” we focused on ”Actual System Usage” with regards to the 360QS technologies that were deployed during the obesity trial. Combining the actual 360QS system usage with the demographic data of the users would allow us to better understand how 360QS could be used for this type of population. In order to evaluate ”Actual System Usage”, we can rely on the amount of data captured by individuals as an indicator of system usage.

### Childhood Obesity

Obesity and the lack of physical activity are the major factors leading to the rise of non-communicable diseases such as diabetes. Childhood obesity is a global epidemic that affects both developing and developed countries [1, 2]. Obesity among children is especially worrying, since the impact on healthcare costs can be a major burden in the future. Furthermore, obesity has been found to increase the risk of a host of health conditions (e.g., sleep apnea, diabetes, cancer, or stroke) [2, 6–8] and also reduce mental well-being (e.g., leading to low self-esteem) [9].

Obesity is a highly complex condition where genetics, environment, education, socio-economic status, climate, schools, and many other factors play a role. Each of these factors can affect individuals differently. Ideally, any health intervention to tackle childhood obesity should take into account all aspects to maximize its chance of success. However, it is not always possible to obtain information about the environment of the affected youth. Furthermore, asking the patient is not always a feasible solution. For example, there is evidence that self-reported questionnaires for behaviors such as sleeping habits can be less reliable than data obtained from wearable devices [10].

For these reasons, designing interventions aimed at weight loss and physical activity promotion is a challenging task [11]. Many external factors (e.g., public transportation, diet, culture, family, schools) can positively or negatively affect the efficacy of public health interventions. Environmental contexts heavily affect decision making; for example social networks have been found to influence obesity [12] both positively and negatively. Another important factor to consider is the temporal aspect of when health decisions are made.

### Quantified self for personal health

Information about personal health and lifestyles can provide insights to patients and healthcare professionals to help them in decision making. Studies have been conducted to provide health behavioral change interventions to promote physical activity at the right time (i.e., Ecological Momentary Interventions) using mobile technology [13].

Personal health tracking is not new. A population survey in the USA found that most American adults keep track of at least one health parameter (e.g., weight, diet, or physical activity) [14]. Related to the behavior of tracking our own health is the concept of Quantified Self. In a 2009 paper, M. Swan defined Quantified Self (QS) as: Quantified self-tracking is the regular collection of any data that can be measured about the self such as biological, physical, behavioral or environmental information” [15]. The rationale behind QS is that quantifying aspects of a daily life through maintaining journals or logs that can help the individual (or the caregiver) to better understand hidden patterns. For example, patients suffering from migraines have been advised to keep diaries of daily activities to identify triggers. Other common examples are food logs, menstruation diaries, growth and vaccination charts for children, and so on. Quantified Self is also present in social media, where people share their weight diaries [16], sleep logs [17], blood glucose levels [18], photos about eating preferences [19], etc. The Quantified self-movement in the health domain is also often part of a social context [20].

Sensors and wearable technologies are sources of data for Health Quantified Self [21]. In recent years the production costs of wearable devices have reduced drastically as they became major consumer products, especially in the fitness and health sector [22]. In parallel, smartphones with multiple sensors (e.g., accelerometers or light sensors) have become mainstream. Moreover, implantable devices are often integrated with wearable devices, such as implantable glucose meters. Also mobile devices are integrating data from smart homes and Internet of Things devices such as smoke detectors. These developments have all led to the Quantified Self movement becoming more mainstream and at a point where incorporating these technologies within the health domain. The popularity of QS technologies for fitness and wellness is paving the way for the development of health applications to support health behavioral change. This also highlights the need to study the use of QS to support behavioral change in populations such as overweight children.

The efforts towards the creation of data-driven holistic views of the individual are reaching unprecedented levels as part of the precision medicine and Big Data for Health initiatives [23]. For example the Kavli HUMAN Project is an effort to aggregate health-related data from 10,000 individuals in New York City containing health, education, genetics, environmental and lifestyle information that will be collected over a period of 20 years [24].

## Methods

The 360QS technology was tested within a research project for childhood obesity in Qatar called ”Adaptive Cognitive Behavioral Approach to Addressing Overweight and Obesity among Qatari Youth” (ICAN Study) [4]. The project was led by Qatar University and consequently received ethics approval from the Qatar University Institutional Review Board. In addition, all participants and their parents/guardians, provided informed consent prior to participation.

Table 1 lists the timeline and processes involved in the ICAN study. The 360QS technologies were deployed in the intervention group from January to August 2016 but not all of the participants consented to using the provided 360QS technologies. Therefore, in this paper, we focus on the subset of participants from the study that interacted with the 360QS technologies and the feasibility of using such technology.

**Table 1:** Timeline of the ICAN Study

Date	Module	Process Description
09/2015	Recruitment	Students list with physiological data obtained
10/2015	Recruitment	Overweight/obese kids selected for study
10/2015	Recruitment	Parents contacted for information session
11/2015	Recruitment	Study information session; consent forms given out to parents
01/2016	Intensive Camp	Health camp begins (Food photos collected)
02/2016	Intensive Camp	Health camp ends (Phones with Instagram, Activity trackers given)
02/2016	Weekend Clubs	Weekend clubs begin
05/2016	Weekend Clubs	Weekend clubs end
06/2016	Summer Break	WhatsApp intervention for mothers begins
08/2016	Summer Break	WhatsApp intervention for mothers ends

### Demographics

Overweight and obese children from several male and female Qatari schools, in the age range of 9 to 12, participated in the interventions. The schools were randomized between intervention groups and control groups. There were 53 boys and 55 girls in the intervention group while the control group consisted of 60 boys and 59 girls.

### Health intervention

The health intervention consisted of a set of health coaching and education actions as follows:

- Health camp: two-week weight management camp from January 24 to February 4, 2016, which included physical and social activities, lifestyle coaching and nutrition counseling.
- Weekend clubs: ten-week program from February 20, 2016, where every weekend for four hours, participants received further reinforcement to acquire healthy behaviors.
- Summer break: during the summer break (June - August 2016) a group of mothers participated in a mobile intervention using WhatsApp to reinforce healthy eating and behaviors.

### The 360QS implementation

The 360QS implementation in the ICAN Study involved different technologies:

- Wearable devices: Wearable devices (actigraphy sensors) were used to monitor sleep and physical activity of the participants.
- Mobile Social Media (Photos and Messaging): We incorporated the use of social media for tracking dietary habits of participants (Instagram) and health education of mothers (WhatsApp).
- Health camp food Photos: We also captured photos of the food consumed by participants during the two-week health camp to gain insights on dietary preferences.
- Physiological Data: Physical health of the participants was measured periodically via height, weight, etc. to obtain an overall picture of the impact of the intervention on participants’ health.

The wearable devices and mobile social media are clear examples of QS technologies that can be used to capture data about individuals’ behaviors. The health camp food photos and physiological data was not captured by the individuals, but the research team. The food photos can be combined with the other data sources to gain more insights on the health behaviors of the individuals.

**Fig. 1:**
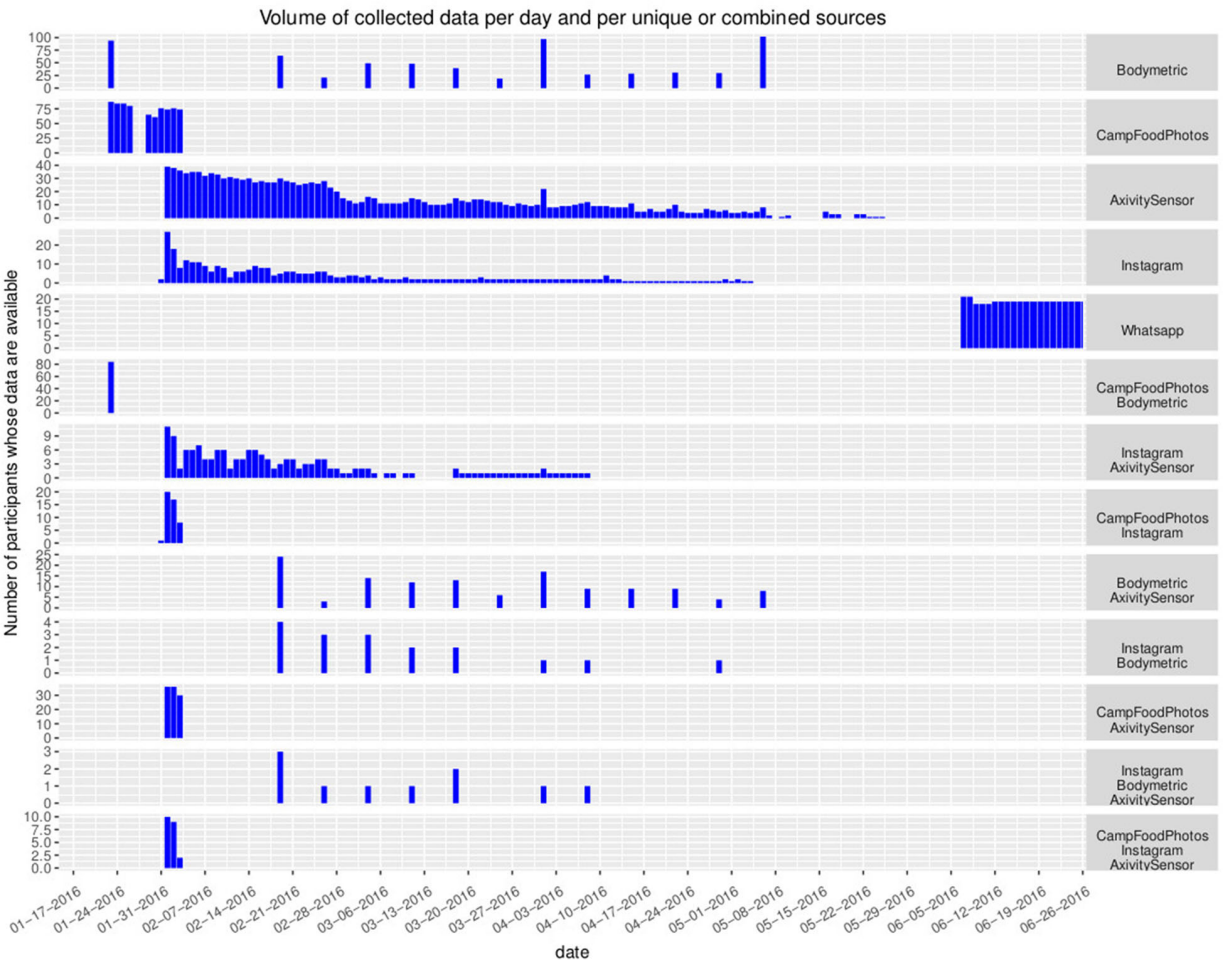
Datasets overview for the 360QS implementation - Volume of collected data from different sources per day. The volume is given in terms of number of participants from which data have been collected for a given day. Each row shows the volume from unique data sources (top five rows) and from combinations of two or three data sources (bottom rows). The combinations of sources are useful to show those data sources for which data is available for the same participants so the complementarity between various data sources can be analyzed in these cases

Figure 1 shows the data collected over time for the 2016 trial using each 360QS technology. It also indicates the time periods with data overlaps. For example, between January 31, 2016 and February 4, 2016, for about 10 participants, we have food photos collected during the camp, physiological data, as well as actigraphy sensor data. As explained below, we need to be aware that these technologies were not applied homogeneously in all the subjects. Not all the mothers accepted to participate in the WhatsApp intervention, and only a subsets of subjects received mobile phones for taking food photos using Instagram.

All the data collected during the trial was integrated using anonymized participant identifiers and stored in a secure database. This integration facilitated the study and analysis of the data.

### Physiological data

As part of the clinical research protocol, all the participants’ physiological parameters were measured over time. That includes weight, height, body mass index (BMI), body fat percentage, waist circumference and blood pressure. These parameters were taken during recruitment, start and end of the health camp, during the weekend clubs and also at the end of the program.

### Health camp: food photos

**Fig. 2:**
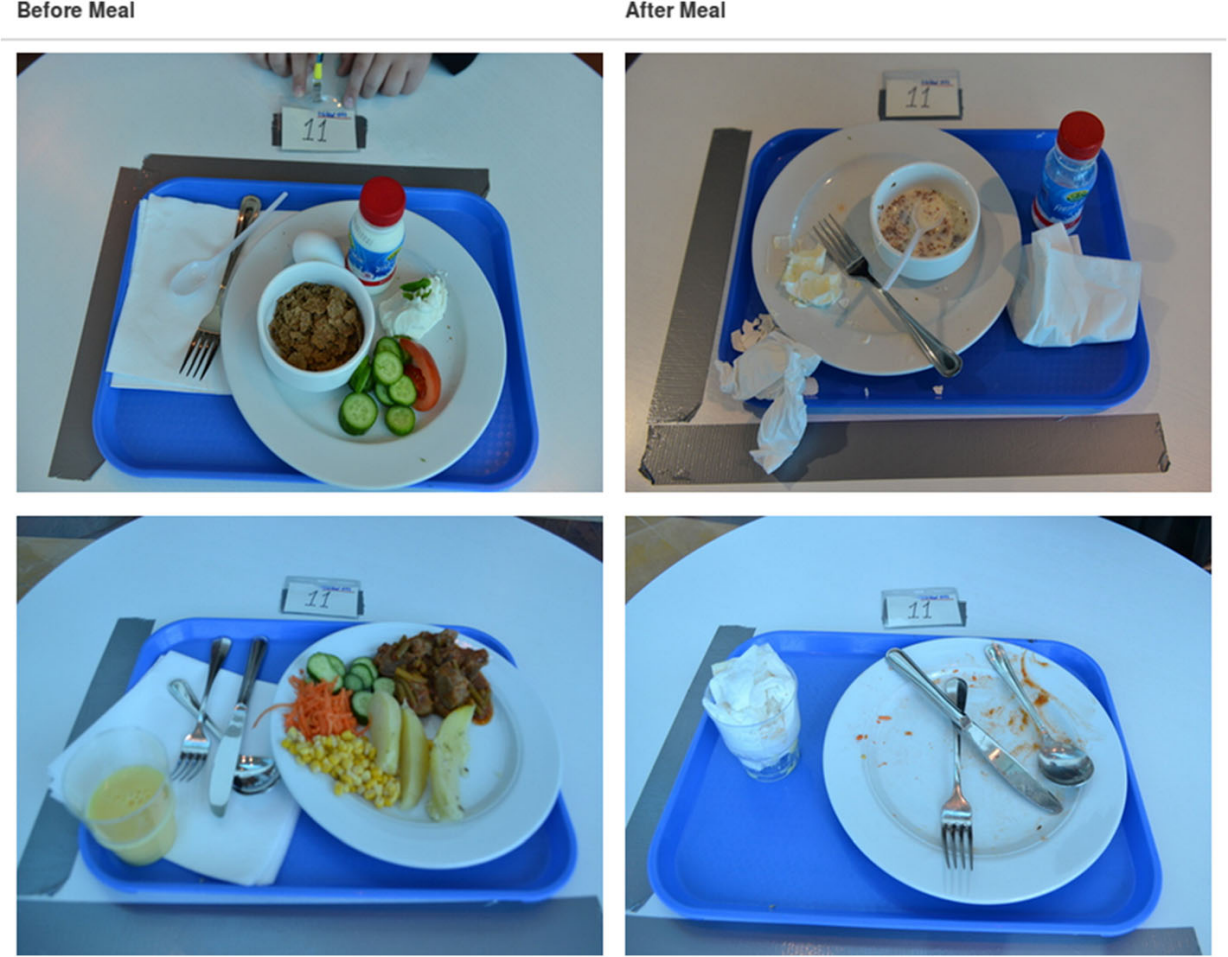
Example of food tray before and after meal

During the two-week health camp, before and after photos of food trays (breakfast and lunch) were manually taken for all participants for ten days, i.e. twenty meals, as shown in Fig. 2. The goal of this data collection was to learn food preferences of participants, which could in turn assist the nutrition professionals in designing diet plans for weight loss, and also give calories estimates of the food consumed during the camp.

### Weekend clubs: actigraphy sensor

In order to study the physical activity and sleep patterns, we used actigraphy. Actigraphy sensors were given at the end of the health camp to be used during the weekend clubs. An actigraphy device is a wearable medical device, which captures motion using accelerometers and other sensors to identify patterns in physical activity and sleep. We used the device Axivity AX3 (see product website: http://axivity.com/product/ax3) that has been used before in clinical studies [25]. The Axivity device is waterproof and was given to participants to be worn at all times as a wristband. The device was given to 67 participants; 16 in the control group (8 boys and 8 girls) and 51 in the intervention group (26 boys and 25 girls). Since the battery life of the device as well as its data storage memory is about four weeks, we recharged the devices and downloaded the data for participants in the intervention group multiple times during the weekend clubs.

### Weekend clubs: social media - instagram

**Fig. 3:**
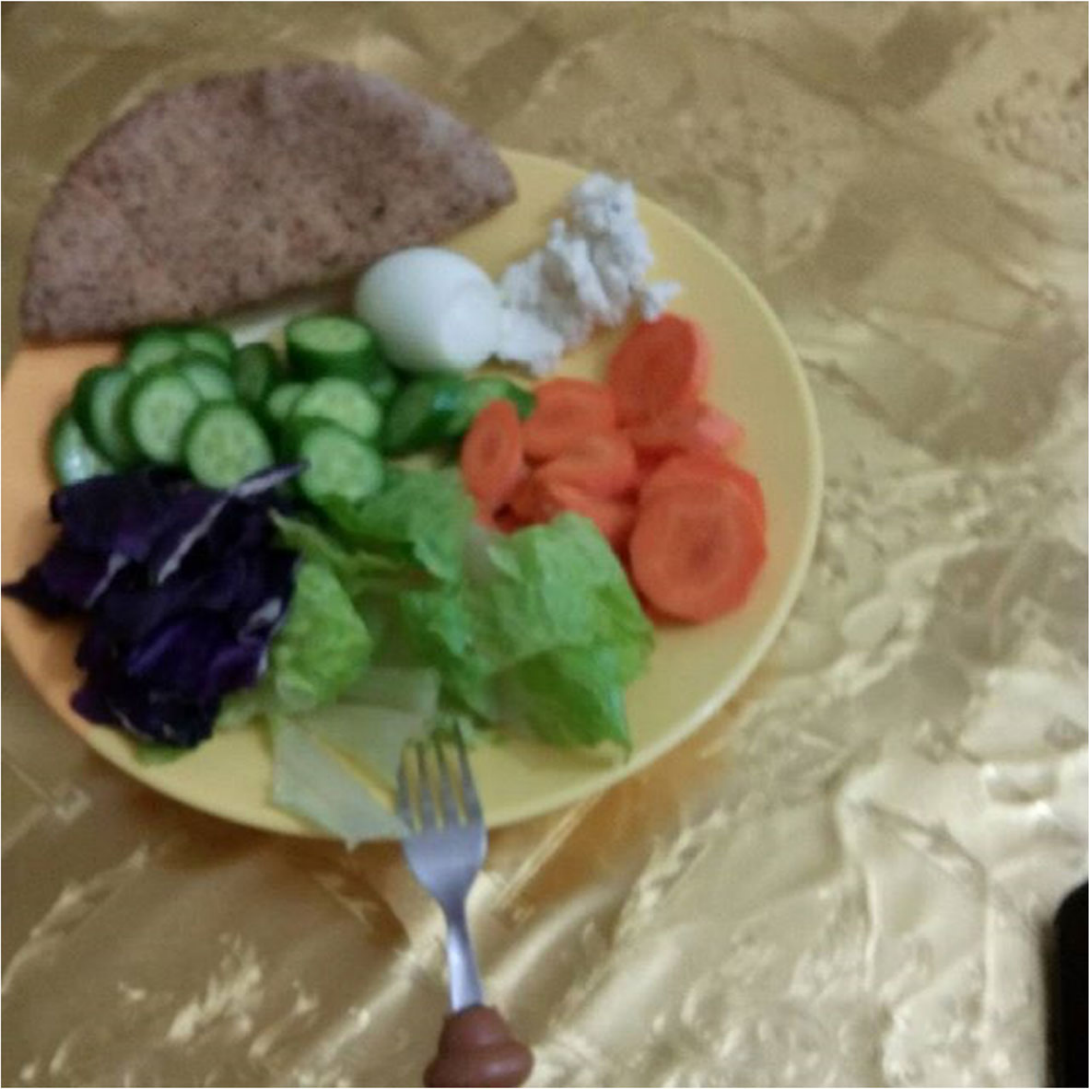
Instagram Photo uploaded by a participant

A total of 50 mobile phones with a pre-configured Instagram account were given to parents of participants (30 boys and 20 girls) at the end of the health camp, to take photos of the food eaten by their children in the remaining duration of the program, and upload to the Instagram account. This was done in order to monitor impact of the health camp on the participants’ dietary habits and also capture dietary preferences of the families. Figure 3 shows one of the participants’ Instagram photo.

### Summer break: social media - WhatsApp

**Fig. 4:**
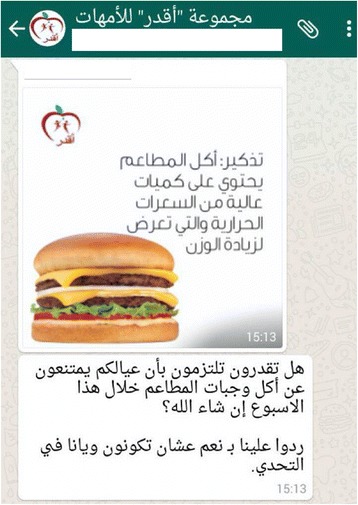
Example of WhatsApp educational message sent by the moderator of the intervention. Translation for message 1:” Remember: restaurant food is high in calories and increases weight.” Message 2:” Can you promise to not let your kids eat out this week? Reply yes if you accept the challenge”

In the ICAN study, face-to-face sessions with parents were organized for health education. Furthermore, we contacted parents asking for their interest in using mobile technology or social media to reinforce the educational support. Only the mothers showed interest in such additional support, with the majority mentioning WhatsApp as their preferred communication channel. We designed an educational intervention for mothers using WhatsApp for a 12-week period of the summer break. Educational content was adapted to the WhatsApp channel (e.g., short educational messages, see Fig. 4). These messages also incorporated the use of behavioral nudges, in particular commitment to support the health changes of their children. The group was moderated by a dietitian and a pharmacist who shared messages that included: daily reminders, weekly healthy tips, weekly commitment for a health change and answers to questions raised by mothers.

A total of 21 mothers were enrolled in this study, with 3 dropouts at the beginning of the study, resulting in 18 participants (mothers of 7 boys and 11 girls) who completed the study. The data collected included the participation in the WhatsApp group, responses to the health tips and also whether the participants had seen the messages or not in order to evaluate the attrition of this intervention.

## Results

### Health camp: food photos

The dataset collected was cleaned and labeled using participant identifier, meal type (lunch or breakfast) and food tray state (before-meal or after-meal), resulting in 2,753 photos. An extensive menu list was prepared by identifying all food items served during the camp. The food photos were grouped into before-meal and after-meal photos per user per day per meal. The labeling tasks were then assigned to local volunteers via a web application where they labeled each task to indicate food items present on the participants’ trays, along with the amount left on the tray at the end of the meal for each food item.

**Fig. 5:**
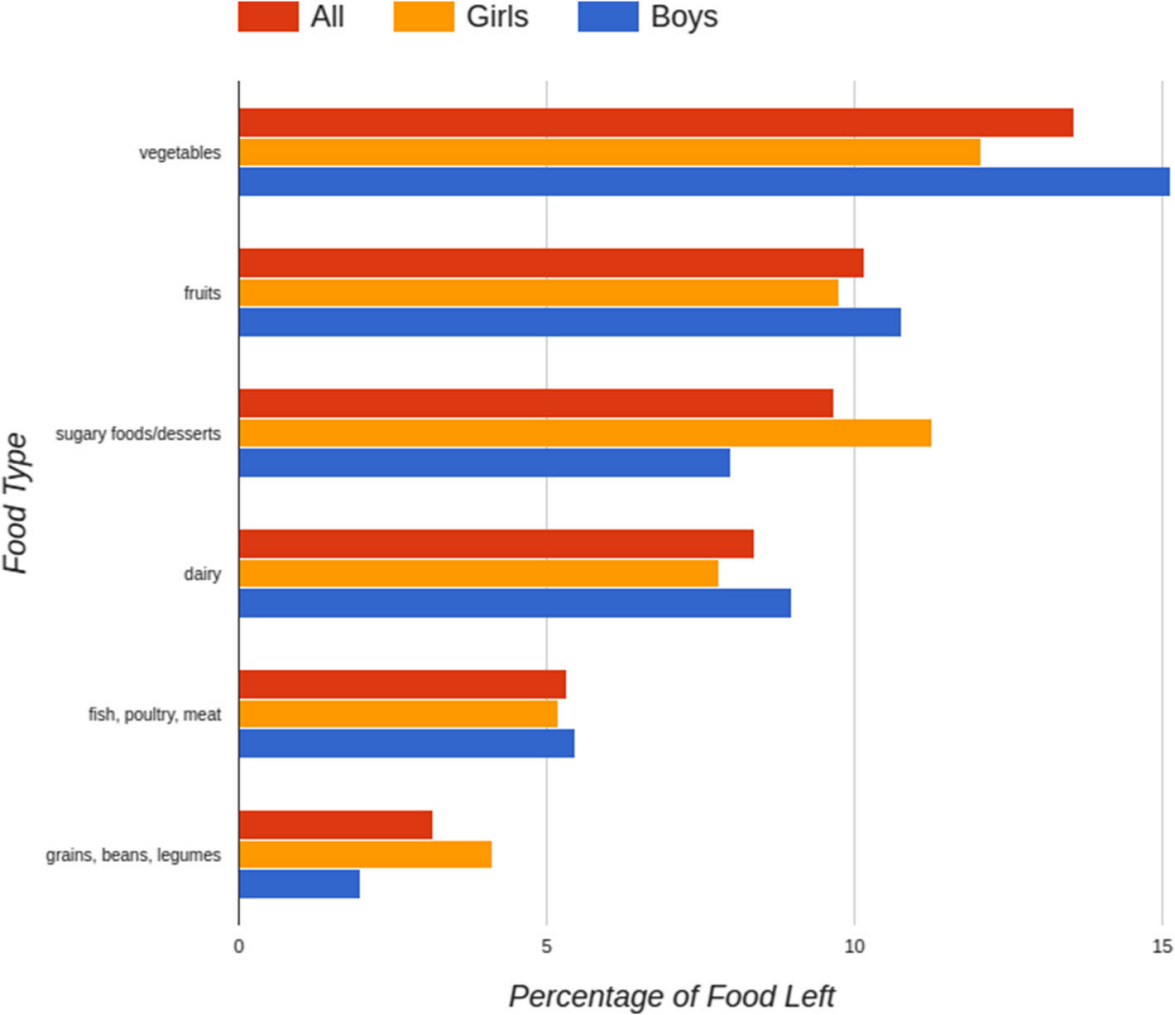
Percentage of food left on trays grouped by food type

Figure 5 shows the percentage of food left on participants trays grouped into six major food types - fruits; vegetables; dairy (milk, cheese, yogurt); fish, poultry, meat; grains and beans/legumes; desserts and sugary food like jam. This data is then separated by gender to get food preferences of girls vs. boys. Vegetables and fruits are the food types most left on trays, where as meat and grains were left the least. This could indicate the food habits of the families of the participants where most meals might consist only of the main course, e.g., meat with rice/bread, with less focus on fruits and vegetables.

**Fig. 6:**
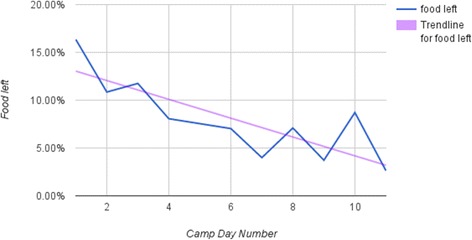
Percentage of food left over all days of the camp

Calculating the amount of food left over the course of the health camp, it is evident from Fig. 6 that more participants started finishing the food they took, indicating either that they stopped taking foods they realized they did not like, or that they got used to all the different food types and increased their intake of the healthier foods as well.

Table 2 shows the correlation of the proportion of foods eaten, grouped by food type at a lower granularity, to the change in a child’s BMI. Most relationships are not significant. However, the negative relation of porridge and dairy at fairly low p values may point towards the importance of breakfast foods. The BMI change is comparing the values from January (beginning of health camp) with the values from May (the end of Weekend clubs). Therefore, with regards to food photos the BMI just shows an overall tendency with regards to health habits.

**Table 2:** Correlation of the proportion of food eaten, grouped by a food type, to the child’s change in BMI

Group	Correlation	p-value	Permuted p-value
vegetable	−0.055	0.611	0.413
fruit	0.061	0.573	0.943
egg	−0.065	0.559	0.556
porridge	−0.145	0.185	0.021
dairy	−0.122	0.269	0.224
meat	0.027	0.803	0.166
fish	−0.093	0.526	0.767
soup	0.120	0.282	0.983
pasta	−0.084	0.526	0.133
rice	0.225	0.050	0.949
bread	−0.014	0.897	0.296
dessert	−0.141	−0.141	0.227

### Weekend clubs: actigraphy sensor

The actigraphy sensor was given to 51 participants. Normally these sensors are given for periods of one week to do an assessment of the levels of physical activity and sleep patterns. In our study we opted to ask the participants to wear it for a longer period (a total of 12 weeks) in order to be able to monitor changes in physical activity and sleep during the health interventions. As expected the adherence declined overtime, and 21 devices got lost.

**Fig. 7:**
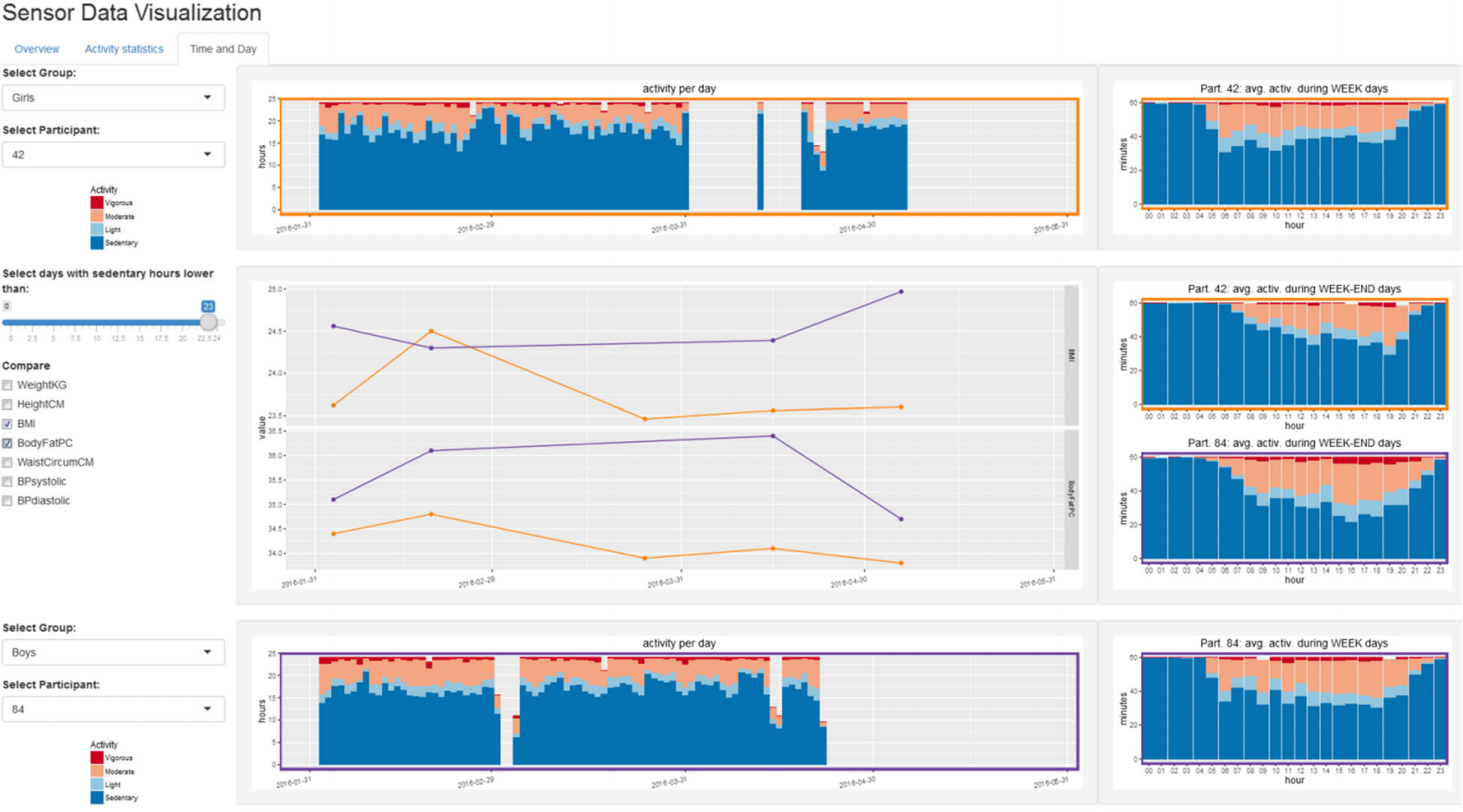
User interface of the visual analytic tool for actigraphy sensor data The interface allows comparing two participants’ activity level (left side) for the full time period and on daily-based average during week and weekend days (right side). Specific clinical measurements like BMI or body fat can also be compared during the same time period (line charts)

Figure 7 shows the visual analytic tool we developed to explore the actigraphy sensor data. It shows the level of physical activity recorded by the actigraphy device through time for all children. It also shows the total activity for boys and girls separately and allows getting detailed activity averaged per day in a user-selected time window.

Relating the activity levels to the change in the child’s BMI during the study period, we compute Pearson product–moment correlation coefficient r at different levels. The raw average number of minutes logged per day did not have a statistically significant relationship with the change in BMI. However, the number of active days, defined as days in which active minutes is non-zero, correlates with BMI change at *r* = −0.263 (*p* = 0.101), meaning the more days are recorded as active, the more negative the BMI change is. The relationship becomes less strong as we define active days more strictly: more than 30 active minutes per day results in *r* = −0.201 (*p* = 0.214), and more than 4 h *r* = −0.140 (*p* = 0.377). This suggests that mere adherence to wearing of the device correlates with beneficial health outcomes. This however might be caused by increased engagement of wearable device users, not necessarily implying causality.

### Weekend clubs: social media - instagram

**Fig. 8:**
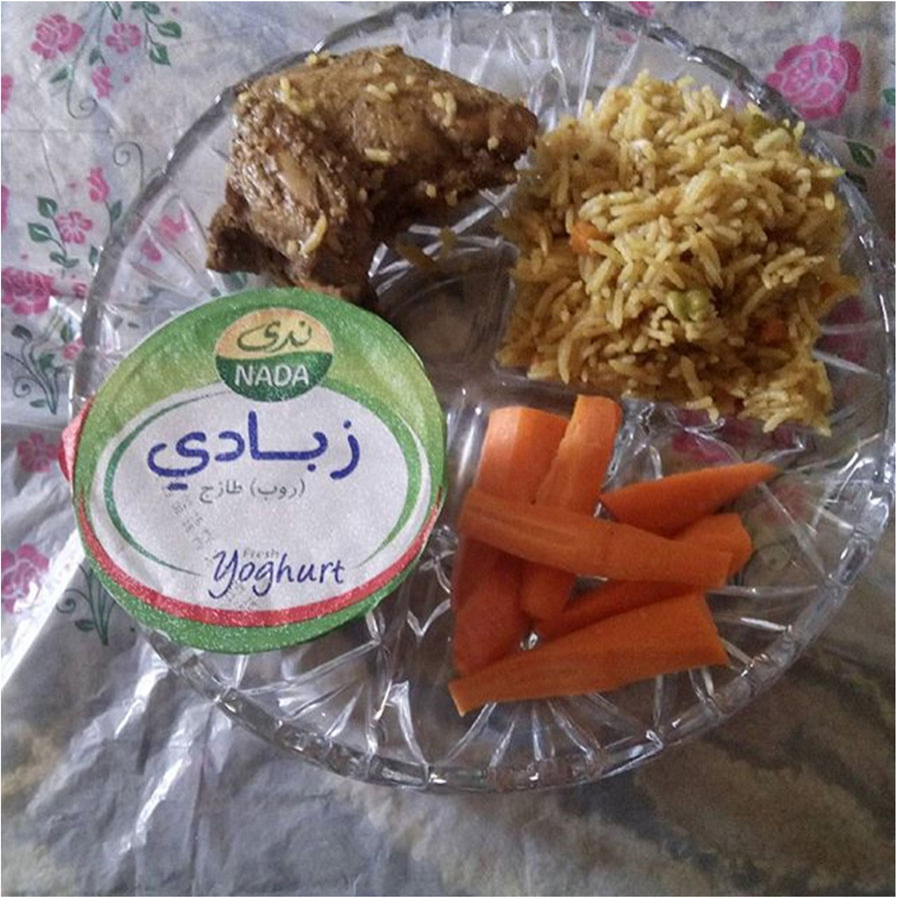
Instagram posts uploaded by users

Of the 50 mobile phones with a pre-configured Instagram account, only 25 were used for such purpose. The number of photos acquired was 937 in total, but the top 3 contributors (1 girl and 2 boys) uploaded 70 percent of the photos, as shown in Fig. 8.

Correlating the extent of use of this Instagram account to the change of child’s BMI results in *r* = −0.179 (*p* = 0.425). To overcome the low p-value due to sparsity, we employ a permutation test to test whether the relationship is significant compared to a randomly permuted outcome variable (here discretized to below/above average BMI change). This approach results in a permuted *p*-value = 0.026. Again this shows that more active users tended to slightly have better health outcomes but that this is most likely related with a higher level of engagement in their health.

### Summer break: social media - WhatsApp

A total of 18 mothers participated in the WhatsApp support group after the Weekend clubs. This was done to provide educational support during the summer break. We analyzed the already acquired weight loss at the time of enrollment in WhatsApp.

That BMI change is comparing the values at beginning of health camp with the values at the end of Weekend clubs. The children of participating mothers got an average BMI change of −0.058 compared to 0.0455 for those not participating (the different was not significant with *p* = 0.7879 and permuted *p* = 0.5709). This indicates a bias where more engaged mothers in terms of health are also more likely to opt-in for the enrollment in this social media intervention. The interaction declined overtime with regards of messages sent in the WhatsApp group (146 in June,108 in July and 23 in August). An important topic during June was the Holy Month of Ramadan and also its impact on nutrition (e.g., big family meals during evenings, fasting). It is common in Qatar that families travel abroad during the summer holidays, especially August, which might have also influenced mobile usage and resulted in reduced interactions.

June was also the month with more questions from the participating mothers. Only four participants did not send questions or confirmations with regards to health tips and comments. Furthermore, 17 out of 18 participants received and read all the messages during the intervention. Only one dropout happened during this period, which was a month after the Whatsapp group started.

## Discussion

### Principal Results

#### Human factors and acceptability

In order to minimize the participant burden, we did not perform questionnaire-based usability evaluation and rather focused on actual system usage. Instead we looked into the attrition towards the use of the different types of technology. Then our findings were discussed with the healthcare professionals that interacted directly with the children and their families to gain insights about the potential reasons.

We found significant differences with regards to the participation and acceptability across the genders. Overall, the engagement of the females was higher. Girls used the wearable devices much more as instructed. Mothers did enroll in the social media intervention where as not a single father showed interest in participating. Moreover, mothers of girls were the majority in the Whatsapp group. This highlights the importance to take into account gender aspects when implementing eHealth solutions to reduce attrition.

Cultural acceptability is a crucial human factor to consider. The design of the WhatsApp intervention had to be adapted to the religious and cultural traditions with special emphasis on the Holy Month of Ramadan. Nutritional advice had to take into account the religious month, which includes some aspects that can vary from family to family (e.g., at which age children start fasting). The retention rates of the mothers in this group were quite high (e.g., few dropouts, nearly all the content read), but participation declined after Ramadam and during school holidays. Further, the WhatsApp intervention was designed only for mothers, as it is not culturally acceptable for many to have mix-gender interactions even in social media. Not a single father showed interest in participating in a social media group.

We noticed a tendency in which the use of technology correlated with lower BMI. We need to be very cautious about inferring causality though, since it is more likely to be a result of engaged children and families being more supportive of technological enhancements for the intervention. The design of our study does not allow us to drawn conclusions about the efficacy of the use of technology for the behavioral change. Future research will need to look at which technologies are more likely to be accepted by wider number of participants.

The healthcare professionals involved in the weight loss camp informed us that gender issues also affected the differences in terms of self-efficacy across genders. This might also explain why girls used the QS technologies more than the boys. Overall, there is a need to study in deeper detail the psychosocial factors that are influencing technology acceptance for health behavioral change among Arabic youth. Our study can only highlight differences, but not the reasons behind the same. In this feasibility study we had continuous conversations with the health team involved in the intervention, who are also co-investigators in this study. Our findings helped them make better decisions while providing health coaching to the participants of the study. As this is a feasibility study about the 360QS data collection, we did not have ready a tool that can assist them during the decision making process. Consequently, our study did not have an impact in the quality of the health intervention. In order to do that, implementation research will need to be performed to identify ways in which the 360QS can be incorporated in the workflow of a health camp such as ICAN. As explained below, visualization of the data will be a crucial aspect.

### Technical challenges

There are complex challenges in statistically analyzing temporal and heterogeneous data. Measuring causality from an intervention is not trivial, despite access to diverse heterogeneous data [26]. Moreover, integration of such datasets, is itself a complex challenge [27]. One of the main challenges is to aggregate highly heterogeneous data into a single platform.

We conducted meetings with the healthcare professionals involved in the trial in order to get their feedback about the utility of the different Quantified Self technologies tested in the study. They highlighted that to integrate such technologies in the practice it is very important to create tools to visualize trends and gain insights about the behaviors of the participants. In terms of visualization, it requires careful construction of the interface to focus on the user needs, providing only the information required for the current analytic task. Interaction is key for understanding, so graphical elements and codes must be linked throughout the interface in a coherent manner. To handle large-scale data, especially coming from actigraphy sensors, it is necessary to aggregate data by time periods like days or hours, to segment activity patterns using automatic techniques, and to give details only on demand as standard according to the visualization mantra “Overview first, zoom and filter, then details-on-demand.” [28].

Accessing the useful data for a given analysis is as important as analyzing the selected data themselves. So another challenge is to give a legible visual overview of the available data for all and each individual, in terms of the data type (photos, messages, sensor data, physiological data) and number of data records, throughout the entire time range. After providing an overview, the tool should allow for a selection of specific records given the type of analytic task requested by the user.

The collected data can be useful to healthcare providers to analyze children profiles and guide them to improve their health (for instance determining which food is preferred, or at which time a child is willing to be active and using this knowledge to orient the child’s behavior); but also for researchers to improve on the measurement protocols and techniques (for instance how to make children more diligent in wearing or using the devices? how to develop analysis techniques to extract more relevant information from the data?); and finally for the children to keep track of their progress in a specific attractive user interface (shall the children be put in competition with each other? or only aware of their own results? or be put in situation to help each other? what kind of information to show to children? and in which way to make it attractive for them?). Each of these needs require specific visual interfaces carefully designed in close collaboration with the targeted end-users and professional experts in children health, nutrition, pedagogy and psychology.

### Comparison with prior work

To our knowledge this is the first reported exploration of the use of Quantified Self for personal health in an Arab country. Our study is highlighting many of the societal challenges that can be faced in introducing digital health interventions in the younger Arab population. There are previous studies in the region, but they have focused on a much narrower implementation of eHealth technologies [29] and specially focusing on diabetes and obesity in the adult population [30, 31]. Another novelty of our study is the combination of social media as an additional source of health quantified-self data. This is increasingly important since consumers are using social media for health purposes. There are other projects looking into the integration and visualization of Quantified Self health data [32, 33], but do not include the heterogeneous datasets as we have.

Apart from heterogeneity, our datasets have an added complexity of being sparse. Sparsity of the data poses significant challenges for visualization. This data sparsity can be attributed to human factors since we do not address visualization of data for people adherent to the ‘Quantified Self’ movement but rather individuals who are in need of a more active role in their health. This is a substantial difference if we consider that many Quantified Self users are highly motivated individuals [20] and that they do not represent the vast majority of people in need of support for behavioral change.

An additional challenge that we describe is how to integrate that information into meaningful visualizations that can help understand the health patterns of the individuals. This is not answered in our paper, but we provide some innovative examples of visualization of this type of data. There are other studies looking in the visualization of Electronic Health Records (EHR) for helping decision making [34], also including Quantified Self data [35–37] but in those studies the QS data is limited to wearable sensors data. Folter et al. studied the integration of health habit data (e.g., motion data for physical activity) to help decision making of healthcare professionals [36]. Ledesma et al. studied techniques for visualizing personal health data, but not including social media data. In our case we are looking into helping understand health behaviors (and not clinical aspects) in a more heterogeneous and sparse data set. Implementation Research methodologies [38–40] can provide a good framework for future work about the integration of 360QS in this type of health interventions.

### Limitations

The implementation of the 360QS was not integrated in the workflow of the health intervention; therefore we have not studied how such an ecosystem can be implemented in a way that can enhance the intervention. Further research should look into the best strategies to use the most promising 360QS technologies as part of the regular intervention.

The clinical trial was registered after the recruitment. However, in this study we do not look into the health outcomes derived from the use of the 360QS ecosystem. Our study design was not implemented to infer health outcomes that would have required a very different experimental setup. We looked at statistical significance of health outcomes for descriptive purposes, overall the p-values were low and the mere fact that some correlation values are small is quite positive if we consider the very small sample size and the presence of multiple confounding factors.

Our study focuses on 9 to 12 years old children, and consequently our findings might not be generalizable to the entire age spectrum of childhood obesity. Further, our study has taken place with Qatari public schools where the medium of education is Arabic. Consequently, our findings might not be applicable to the non-arabic speaking segments of the population in Qatar.

In order to be able to understand the reasons for low usage of some of the 360QS technologies, we will need to perform qualitative studies (e.g. questionnaires, semi/structured interview) addressing different aspects of the Technology Acceptance Model [5] and Human Factor Engineering methods. Further, ways to visualize the 360QS data to help decision-making were not addressed in this study and it is part of future work.

## Conclusions

In this study we explored the feasibility of using the 360° Quantified Self technology to capture health-related data within a childhood obesity intervention. The integration of mobile, social media, wearable and health data is feasible but there are many potential challenges related to human factors and technical issues (e.g. gender differences in technology usage). It is not yet clear if the increased adherence to technology was more prevalent among motivated children and families, and this might affect the representative data derived from 360QS. We proved the feasibility of using 360QS in childhood obesity studies to capture health-related data, but in order to implement it, careful planning and integration in workflow of the health professionals is needed.

## Abbreviations

360QS: 360° Quantified Self
BMI: Body Mass Index
EHR: Electronic Health Records
GCC: Gulf Cooperation Council
QS: Quantified Self
