# Erratum to: Implementing 360° Quantified Self for childhood obesity: feasibility study and experiences from a weight loss camp in Qatar

**DOI:** 10.1186/s12911-017-0457-x

**Published:** 2017-05-12

**Authors:** Luis Fernandez-Luque, Meghna Singh, Ferda Ofli, Yelena A. Mejova, Ingmar Weber, Michael Aupetit, Sahar Karim Jreige, Ahmed Elmagarmid, Jaideep Srivastava, Mohamed Ahmedna

**Affiliations:** 1 Qatar Computing Research Institute, Hamad bin Khalifa University, HBKU Research Complex, Qatar Foundation, Education City, Doha, Qatar; 2 Department of Human Nutrition, College of Health Sciences, Qatar University, Doha, Qatar

## Erratum

Upon publication of the original article [1], the incorrect figure was included as Fig. 8. Additionally, the following paragraph found in Results, under the subheading; Weekend clubs: social media- instagram “The number of photos acquired was 937 in total, but the top 3 contributors (1 girl and 2 boys) uploaded 70% of the photos, as shown in Fig. 8” should read “ The number of photos acquired was 937 in total, but the top 3 contributors (1 girl and 2 boys) uploaded almost 70% of the photos, as shown in Fig. 8” This is to reflect that the actual percentage of 66.5% was rounded to 70%.

**Fig. 8:**
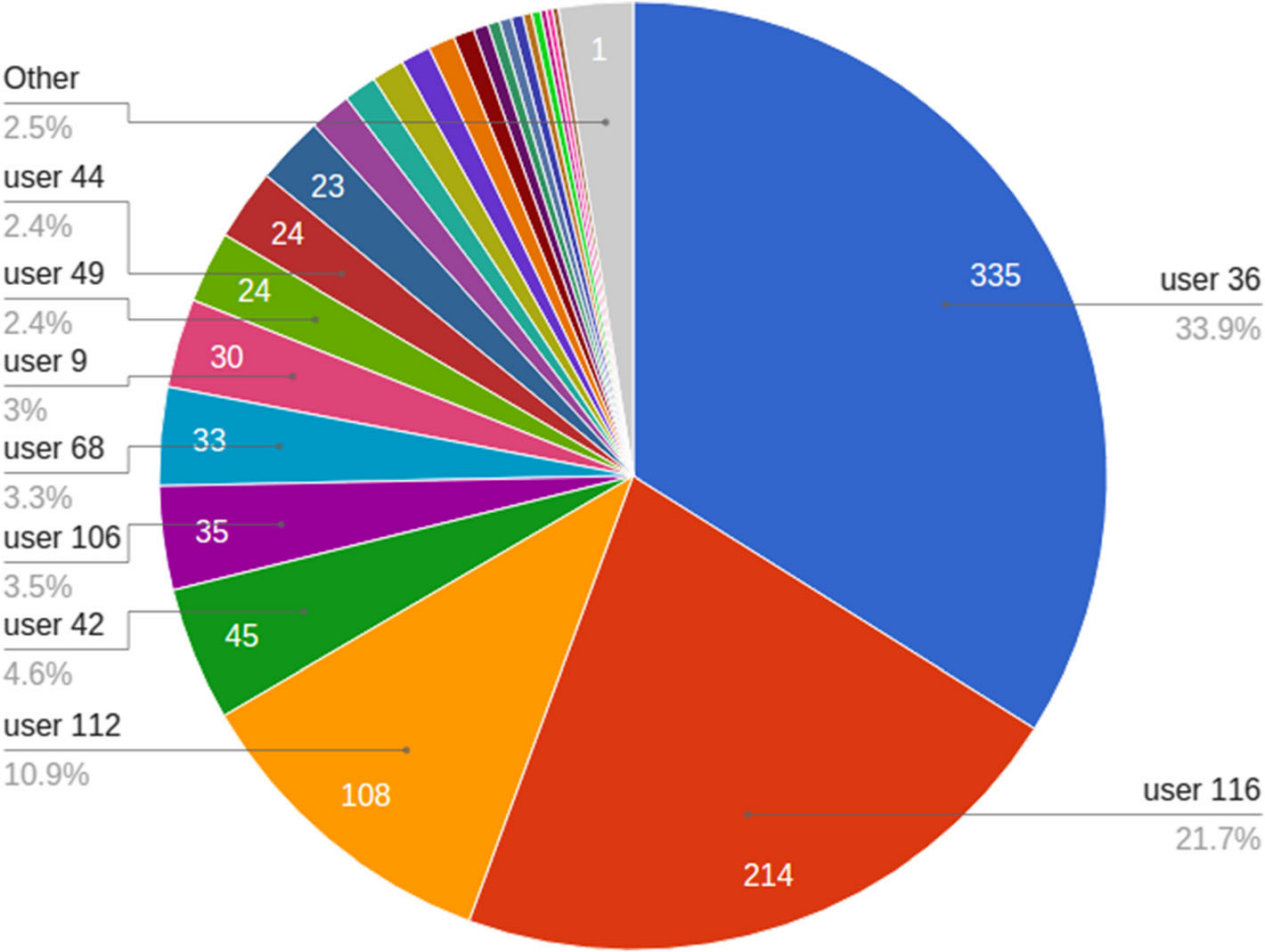
Instagram posts uploaded by users

This has since been acknowledged and corrected in this erratum.
